# *Notes from the Field*: Use of Asynchronous Video Directly Observed Therapy for Treatment of Tuberculosis and Latent Tuberculosis Infection in a Long-Term–Care Facility ― Puerto Rico, 2016–2017

**DOI:** 10.15585/mmwr.mm6650a5

**Published:** 2017-12-22

**Authors:** Henry Olano-Soler, Dana Thomas, Olga Joglar, Katrina Rios, Milton Torres-Rodríguez, Greduvel Duran-Guzman, Terence Chorba

**Affiliations:** ^1^Public Health Associate Program, Office for State, Tribal, Local and Territorial Support, CDC; ^2^Division of Tuberculosis Elimination, National Center for HIV/AIDS, Viral Hepatitis, STD, and TB Prevention, CDC; ^3^Tuberculosis Control Program, Puerto Rico Department of Health; ^4^Division of State and Local Readiness, Office of Public Health Preparedness and Response, CDC; ^5^Emocha Mobile Health, Inc., Baltimore, Maryland; ^6^Johns Hopkins University School of Medicine, Baltimore, Maryland; ^7^Division of STD/HIV Prevention, Puerto Rico Department of Health; ^8^Central Office for HIV/AIDS, Viral Hepatitis, STD and TB Prevention, Puerto Rico Department of Health.

To treat a cluster of tuberculosis (TB) transmission cases in a long-term care facility for cognitively impaired adults located in Puerto Rico (facility A), the Puerto Rico TB Control Program used a novel video directly observed therapy (VDOT) application. In 2016, active TB disease was diagnosed in 11 residents and latent TB infection (LTBI) was diagnosed in six residents of facility A. Asynchronous VDOT was used to monitor treatment for these 17 residents. One of the patients with active TB disease had received a diagnosis of LTBI during an investigation at facility A during 2011–2012.

During 2010–2012, seven residents of facility A received a diagnosis of active TB disease; four of these diagnoses were culture-confirmed, with isolates that had the same rare genotype (*1*). Drug susceptibility testing indicated sensitivity to the standard first-line regimen of rifampin, isoniazid, pyrazinamide, and ethambutol (RIPE). Three of the seven TB patients died before starting treatment; the other four were prescribed the RIPE regimen under the supervision of personnel from facility A. Two of the four patients who reportedly completed RIPE treatment in 2012 died in 2016 from unrecognized TB-related conditions; both patients were roommates of the 2016 index case patient. For these two patients, evidence of TB discovered during a postmortem medical record review included ineffective antibiotic treatments for putative community-acquired pneumonia and bronchitis and signs of wasting, which were corroborated by interviews with staff members and treating physician. No patients at facility A tested positive for human immunodeficiency virus infection in 2012 or 2016. The contact investigation performed in 2011–2012 identified LTBI in 26 residents and seven nonresidents. All contacts with LTBI were reported by facility staff members as having completed treatment with 4 months of daily rifampin (4R), one of a few standard LTBI regimens, in 2012.

On June 20, 2016, a resident of facility A, who was a contact from the 2011–2012 investigation and whose facility records indicated prior treatment for LTBI with 4R, was identified as having advanced cavitary TB disease; the genotype and drug susceptibility testing of this patient’s isolate matched that of the original cases. This resident began treatment with a 6-month course of RIPE; ethambutol was discontinued after drug sensitivities were confirmed. Among 38 residents and 15 staff members, 10 additional cases of active TB disease were diagnosed among residents; these patients were prescribed rifampin, isoniazid, and pyrazinamide (without ethambutol). Six other residents with diagnosed LTBI were prescribed 4R treatment. Because of staffing shortages, Puerto Rico Department of Health (PRDH) TB field personnel were not available to administer daily directly observed therapy (DOT) at facility A and facility A did not have the personnel needed to provide daily patient transport to the PRDH clinic.

VDOT uses video and computer equipment that allows public health officials to observe patients taking medications for TB, and it has been successfully used to ensure proper completion of TB treatment (*2*–*5*). A standard live VDOT protocol (e.g., using FaceTime) (*4*) was attempted at facility A but was not sustainable because cell phones or Internet connectivity were not consistently available. An asynchronous VDOT protocol that did not require real-time Internet connection or a cellular plan, complied with the Health Insurance Portability and Accountability Act, and provided a Spanish external-facing application*^,†^ was implemented to ensure proper treatment for TB and LTBI patients. Use of this asynchronous system avoided audio/visual interruption related to poor connectivity, which can be problematic in standard live VDOT applications (*4*), by capturing and storing videos of patients as they swallowed their TB medications, and automatically uploading the videos after Internet connection became available. Videos were viewed by PRDH staff members at 2–10 times the speed at which they were recorded. In addition to the clinic-to-facility commute, which would have taken 1.5 hours per day, DOT for the 17 severely cognitively challenged men would have required an additional 1.5 hours per day of observation. Use of asynchronous VDOT saved PRDH approximately 240 hours in DOT-related activities, equivalent to 25% of the workload for a full-time epidemiology technician/case manager over 6 months of treatment*.*

As of July 12, 2017, all 11 patients with active TB disease and all six with LTBI had completed treatment with recommended ≥80% compliance (percentage of scheduled doses actually taken) (Table) (*6*). Active TB disease treatment rates were higher than those for LTBI because protocols exist for treating 5 days per week; LTBI treatment, however, is normally 7 days per week and, in this case, was extended by 1 month to achieve ≥80% compliance. All patients with active TB disease have shown clinical signs of improvement. In addition to using daily symptom queries attached to the videos and telephonic communication as needed, the medical director used asynchronous VDOT to observe directly any complex patients on multiple hepatotoxic drugs for side effects that could interfere with treatment compliance and to verify a daily measurement of treatment completion. VDOT has been demonstrated to be cost-effective in multiple settings (*5*). CDC has developed an eDOT toolkit (https://www.cdc.gov/tb/publications/guidestoolkits/tbedottoolkit.htm) to facilitate adoption of these practices.

**TABLE:** Active tuberculosis (TB) disease and latent tuberculosis infection (LTBI) patient compliance with daily directly observed therapy verified through asynchronous video ― Puerto Rico, 2016–2017

Patient no.	% Compliance*	No. doses taken^†^	No. doses scheduled	Weeks of treatment^§^
Active TB cases (n = 11): completion of 6-month treatment for active TB disease with RIF, INH, and PZA*				
11	94	132	140	28
4	93	124	133	37^¶^
5	91	128	140	28
7	90	126	140	28
8	92	133	145	29
9	96	149	155	31
10	93	121	130	26
12	90	117	130	26
13	91	127	140	28
14	93	125	135	27
15	93	130	140	28
All	92	1,412	1,528	—
**LTBI patients (n = 6): completion of 4-month treatment for LTBI with RIF**				
16	86	94	110	22
17	88	96	110	22
18	88	97	110	22
19	85	93	110	22
20	87	95	110	22
21	91	100	110	22
**All**	**87**	**575**	**660**	**—**

**Abbreviations:** INH = Isoniazid; LTBI = latent TB infection; PZA = Pyrazinamide; RIV = Rifampin.

* Percentage of recommended doses taken.

**^†^** CDC recommends completion of 130-dose treatment during a 5 day/week regimen for active TB disease and compliance is recommended to be at least 80%. Doses taken were counted only during weeks in which ≥4 doses occurred (80% compliance). For LTBI, CDC recommends completion of 120-dose Rifampin treatment during a 7 day/week regimen. Duration of treatment was extended from 16 to 22 weeks to accommodate 5 day/week dosing and achieve 80% compliance. https://www.cdc.gov/tb/publications/ltbi/treatment.htm#treatmentRegimens

**^§^** Including the index case, patient 11, active TB patients began treatment over a range of several weeks as clinical signs and symptoms of disease were identified. Group visits to the TB clinic occurred simultaneously for all patients.

^¶^ Patient 4 received a modified treatment plan for active disease during phase 1. Standard doses were taken 3 days/week instead of 5 days/week because of interactions with other medications.

## Footnotes

* https://www.emocha.com/press/_press/downloads/emocha_SA_Expansion_PR_090315_FINAL.pdf.
